# How Does It Feel to Have One's Psychiatric Diagnosis Altered? Exploring Lived Experiences of Diagnostic Shifts in Adult Mental Healthcare

**DOI:** 10.3389/fpsyt.2022.820162

**Published:** 2022-02-11

**Authors:** Cliodhna O'Connor, Christina Seery, Claire Young

**Affiliations:** School of Psychology, University College Dublin, Dublin, Ireland

**Keywords:** diagnosis, psychiatry, mental health, lived experiences, narrative, interviews

## Abstract

**Background:**

Though the socio-emotional significance of psychiatric diagnoses and the frequency of transitions between diagnostic classifications are widely acknowledged, minimal research reveals how “diagnostic shifts” are subjectively experienced by psychiatric service-users.

**Aim:**

This study investigated how adult service-users make sense of diagnostic shifts and their impacts on one's life.

**Methods:**

Twenty-seven people with self-reported experiences of diagnostic shifts opted into this qualitative study. Virtual narrative interviews invited participants to share their “diagnosis stories.” Interview transcripts were analyzed using narrative thematic analysis to identify common and divergent experiences across participants.

**Results:**

Diverse experiences of diagnostic shifts were related: diagnostic shifts could both promote and undermine clinical trust, therapeutic engagement and self-understanding. The analysis suggested that shared and divergent experiences could be attributed to two dimensions of narratives: participants' *Interpretations of Diagnostic Shifts* and *Diagnosis-Specific Factors*. Regarding the former, analysis produced a typology of three possible interpretations of diagnostic shifts, which were linked with consistently different antecedents, experiences and consequences. The latter dimension captured how experiences of diagnostic shifts also hinged on the unique meanings ascribed to the specific diagnoses gained and lost, particularly in relation to their perceived severity, stigma, personal associations, and related communities.

**Conclusions:**

Findings revealed how diagnostic shifts can be experienced as both traumatic and life-enhancing, depending on their social and subjective context. Understanding the range and predictors of variable experiences of diagnostic shifts is vital for sensitive clinical practice and communication.

## Introduction

Mental health and neurodevelopmental difficulties manifest on a continuum, with considerable individual variability in the severity and expression of symptoms experienced. Yet most mental health science and practice depends on classification of psychological difficulties into discrete diagnostic categories, which typically have poor temporal and inter-rater reliability (1). One byproduct of these categorical systems of diagnosis is the movement of a subset of mental health service-users between different diagnostic classifications over time. While such “diagnostic shifts” are common in clinical practice (2, 3), little research has explored their implications for service-users' lives. This paper offers the first such evidence, drawing on narrative interviews with adults with first-hand experiences of diagnostic shifts.

Research with clinical and community samples shows most psychiatric diagnoses have limited longitudinal continuity (2–6). In practice, this means that during a person's experience of psychological difficulties, a diagnosis once received can transition into a different diagnostic classification or be lost entirely. Many factors can engender diagnostic shifts, including change in symptomatology, differences of clinical opinion, clinical errors and their correction, pragmatic efforts to secure diagnostically-linked resources, and evolutions in clinical knowledge and diagnostic instruments (2). Irrespective of their genesis, diagnostic shifts can redirect clinical trajectories, since diagnosis signals particular prognosis and treatment pathways (7). Yet the potential repercussions are broader than purely clinical: diagnostic shifts may instigate ripple-effects across people's lives more globally (8). Understanding the range of such consequences is key for sensitively communicating and managing diagnostic shifts.

Since times of transition illuminate otherwise inconspicuous aspects of the status quo, diagnostic reforms are also an ideal platform to advance sociological understandings of the ways diagnosis affects personal and social lives (9–11). The potential socio-emotional significance of diagnostic shifts arises from diagnoses' role in shaping the ways that service-users understand and communicate psychological challenges (12). Receiving a psychiatric diagnosis can be a transformative life event; Jutel (13) argues “the naming of the disease can sometimes be more powerful than the disease itself.” Medical sociologists root the emotional potency of diagnosis in its power to trigger narrative, i.e., scrutiny of the causal sequence of events (13). Diagnosis can induce a “biographical disruption” (14), prompting re-examination of one's history to pinpoint the biological, familial or societal causes of the disorder. It also diverts future biographies by proposing new prognosis and management pathways.

Receiving a psychiatric diagnosis can involve both positive and negative socio-emotional impacts: while some welcome diagnoses as facilitating validation and self-insight, others find diagnoses threaten and devalue their self-concept (12, 15). At a social level, psychiatric diagnoses can expose people to stigmatization, but can also offer valued social identities built on communities of similar others offering solidarity and support (15, 16). A key factor differentiating this variability in responses is the specific diagnosis in question. Different diagnostic labels activate distinct associations and stereotypes, which color the experience of being so classified (17–19). For example, being told one's feelings of agitation result from Schizophrenia, Bipolar Disorder or Post-Traumatic Stress Disorder may arouse divergent socio-emotional responses.

Thus, people invest social and emotional significance in the diagnoses they are ascribed, with different diagnostic labels carrying variable meanings. It is therefore plausible that revision of an established diagnosis has broad repercussions beyond clinical settings. A previous study confirmed that in child and adolescent mental health contexts, diagnostic shifts had major practical, social and emotional ramifications for young people and families (8). A revised diagnosis could improve treatment options and therapeutic engagement, but also exclude young people from valued services and foster distrust of clinical professionals. Socially, diagnostic shifts re-classified young people into different social categories, which could either support valued shared identities, or expose the young person to new forms of stigma. Emotional responses were also complex: while some families were relieved at the improved understanding that revised diagnosis brought, others experienced confusion, grief and anger (8). No similar research has investigated how adult service-users experience and interpret shifts in their psychiatric diagnosis.

This lack of evidence on how adult service-users respond to diagnostic shifts is of pressing significance. Systems of psychiatric diagnosis are currently in flux: the long-established categorical approach to diagnosis, implemented through standardized manuals such as the Diagnostic and Statistical Manual of Mental Disorders (DSM-5), is under increasing challenge from alternative formulation, precision and dimensional approaches to clinical assessment (20–22). These ongoing debates should consider not only questions of epistemological or clinical validity, but also the pragmatic implications of diagnostic systems for service-users' lives. Responsible implementation of any systemic change in diagnostic systems requires anticipation of the micro-level repercussions of overturning service-users' established diagnoses. Moreover, beyond any wholesale revision of diagnostic systems, diagnostic shifts already occur in routine mental healthcare, but with no evidence available to inform their management or communication. Optimal delivery of mental health services requires understanding the first-hand experiences of service-users (21). The current study used a narrative interview approach to explore how diagnostic shifts were subjectively experienced by an international sample of adult service-users.

## Methods

### Design

Questions regarding service-user experiences are ideally suited to qualitative research methods, which generate in-depth insight into the range of subjective perspectives on a given phenomenon (23). These perspectives can be analyzed “on their own terms” by employing a critical realist epistemological framework, which considers participants' accounts not in terms of their factual accuracy, but their subjective significance within people's lives. Rather than generalizing findings beyond the specific sample studied, the aim is to explore the commonalities and disparities of experiences within a defined group of people (24, 25). In this study, a narrative approach to conducting and analyzing interviews was employed (26, 27), in recognition of the role diagnosis plays in shaping biographical narratives (13).

### Participants

Participants were recruited using online convenience sampling that circulated mass invitations to opt into the study. Adverts for a study of “lived experience of diagnostic shifts in adult mental health” were placed in social media groups and online messaging boards focused on mental health issues. To diversify recruitment outlets, adverts targeted online networks involving different countries and diagnostic groupings. Stated eligibility criteria included age between 18 and 65, capacity to consent, ability to converse in English, and self-identification as having experienced a change of psychiatric diagnosis. No incentives were offered. Those interested in participating emailed the researcher, who confirmed eligibility, sought informed consent to participate, and arranged an interview time.

Thirty-eight people contacted the research team expressing interest in or queries about the research. Following withdrawal of those who did not meet inclusion criteria or opted not to proceed, twenty-seven people took part in an interview. Twelve participated from the USA, five the UK, five Ireland, two Norway, and one each from Canada, Poland, and the Netherlands. Participant ages ranged from 24 to 56 (average = 33) years. Twenty-one identified their gender as female, four male and two non-binary. Eighteen participants reported employment in diverse occupations, alongside one retired, four unemployed and four student participants. Supplementary Material displays the diagnostic trajectories that participants reported, which included a wide range of mood, anxiety, personality, psychotic and neurodevelopmental disorders. If possible, participants were invited to share available documentary evidence (e.g., clinician letters) to validate self-reported diagnostic histories; 11 participants volunteered documentation confirming their diagnostic narratives.

### Procedure

Ethical approval was provided by the University College Dublin Human Research Ethics Committee. As data collection was international and occurred during the COVID-19 pandemic (February-April 2021), interviews took place using Zoom videoconferencing software^1^. Interviews were conducted by one of two research psychologists trained in qualitative interviewing on sensitive topics. Interviews followed narrative interview procedure (26), which began by asking participants to relate their “diagnosis story” in their own words. When their narration naturally tapered off, the interviewer introduced questions that clarified gaps or prompted expansion on under-developed aspects of the narrative. Interviews lasted between 25 and 73 minutes (mean = 46.5 minutes).

### Analysis

Interviews were recorded, transcribed, and imported into NVivo 12 for narrative thematic analysis (27). A coding-frame containing 41 codes was inductively developed to capture recurring narrative features. To optimize accessibility of this analytic instrument, codes were grouped into superordinate categories (e.g., the codes “actively sought diagnostic change,” “change of clinician/service” and “new symptoms” appeared under “Antecedents of Diagnostic Shifts”). The coding-frame was applied systematically to transcripts using NVivo, with meaningful “chunks” of text tagged with any relevant codes.

To validate the coding-frame, one-third (*n* = 9) transcripts were each coded by two independent analysts (28). NVivo's coding comparison tool computed inter-coder reliability (Cohen's κ) of each code. Results showed the coding-frame met accepted standards (29), with 17.1% (*n* = 7) codes showing “nearly perfect” (κ > 0.8), 41.5% (*n* = 17) “substantial” (κ = 0.6-0.79), 24.2% (*n* = 10) “moderate” (κ = 0.4-0.59) and 17.1% (*n* = 7) “fair” (κ = 0.2-0.39) agreement, and none scoring in the “slight” (κ < 0.2) range. Researchers resolved specific coding disagreements through team discussion and tightened the definitions of codes with “moderate” or “fair” agreement. The final coding-frame was then applied to all remaining transcripts by a single coder. Other quality assurance measures included keeping a detailed ‘audit trail', transparent reporting of analytic steps, supporting analytic conclusions with illustrative data excerpts, and attention to discrepant or minority cases (30, 31).

Once coding was complete, NVivo's Query, Crosstab and Matrix functions were used to discern how codes clustered together and were distributed across the data. These inter-relationships between codes were visually charted using NVivo Concept Maps to develop a thematic structure capturing the range of narratives in the data. The analytic priority was to chart the range of experiences related by participants, rather than estimate their quantitative prevalence in this small self-selected sample.

## Results

The analysis identified two thematic dimensions of participants' narratives: experiences differed according to participants' *Interpretations of Diagnostic Shifts* and *Diagnosis-Specific Factors*.

### Interpretations of Diagnostic Shifts

The analysis identified a typology of three possible interpretations of diagnostic shifts: as “*Misdiagnoses and missed diagnoses*,” “*Evolving diagnoses*,” or “*Proliferating diagnoses*.” These three interpretations were not mutually exclusive: a participant who had experienced multiple diagnostic shifts could employ different narratives to characterize different shifts (e.g., one as a misdiagnosis and another as an evolving diagnosis). Depending on the interpretation applied, consistently different antecedents, experiences and consequences of that shift were narrated (Table 1).

**Table 1:** *Interpretations of diagnostic shifts*: typical narrative elements across the three types of interpretation.

	**Misdiagnoses and missed diagnoses**	**Evolving diagnoses**	**Proliferating diagnoses**
Profile	Clinical error/correction	Gradual evolution	Multiple successive shifts
Antecedents	Routine re-evaluation	New symptoms	Change of clinician/service
	Shift actively sought	Institutional developments	Failure to remove redundant diagnoses
Experiences	Relief	Indifference	Frustration
	Regret	Ambiguity	Occasional epiphany
Consequences	Transformed self-concept	Self-understanding	Demoralization
	Improved treatment	Minimal treatment changes	Treatment disengagement
	Undermined clinical trust	Preserved clinical trust	Skepticism of clinical expertise

#### Misdiagnoses and Missed Diagnoses

##### Profile of the Shift

Numerous participants attributed a diagnostic shift to clinical error and its later correction. This error was framed as either a misdiagnosis (i.e., “false positive” application of an inappropriate diagnosis) or missed diagnosis (i.e., “false negative” exclusion of an appropriate diagnosis).

Cases of misdiagnosis generally involved core symptoms, which were originally attributed to one underlying disorder but later remapped to another.

*it was a swap. Pretty much saying, like I feel that all the symptoms that brought you to your incorrect BPD diagnosis were actually like PTSD, presenting in like similar ways. And that he felt the more accurate diagnosis was PTSD. [P1]* ^2^

Cases of missed diagnosis typically involved symptoms originally being overlooked. This was attributed to numerous factors, including overshadowing by more salient problems (e.g., substance abuse), clinical carelessness, or participants withholding pertinent information (e.g., previous traumatizing events).

*I told her, yeah everything and that's when she first was like, so you've had a lot of trauma […] I can see now how that diagnosis would have never come up, yeah, in any past therapeutic relationship or psychiatric relationship, because they never, I never felt comfortable and also they don't take the time to really find that out. [P13]*

##### Antecedents of the Shift

In some cases of diagnostic shifts involving misdiagnosis or missed diagnosis, the shift was independently initiated by a clinician after routine re-evaluation.

*I had around six or more, maybe more, hours of evaluation […] they kind of took the time to go through all the questionnaires and then I was diagnosed with borderline personality disorder. [P17]*

More frequently, a diagnostic correction was actively sought by the participant. Participants sometimes requested re-evaluation based on a vague sense their previous diagnosis did not fully explain their difficulties. In other cases, participants strongly suspected they qualified for a specific missed diagnosis and lobbied for its application. Some reported a long quest for this diagnosis, involving multiple generalist and specialist services.

*I was going to another psychiatrist, but then still in the public system. I told her that I wanted this diagnosis […] She didn't give it to me, she didn't feel confident enough and specialist enough. So I went to the head psychiatrist in that clinic. He did not want to give me that diagnosis either. He suggested, like did you consider borderline? […] so I did what people told me to which was to find a specialized– specialist who specializes in adult ADHD. […] I was well prepared, because, you know, I've been thinking this about myself for ten years and she gave me a diagnosis. [P4]*

Some participants who believed they had been misdiagnosed requested its formal retraction.

*I went in, like you're prepared for a fight. Like, where I had to be like, I don't think I have it, and this is my reason why, here's my slideshow, and like testimonials from like my friends. [P6]*

Participants recalled trepidation prior to clinical encounters where they challenged their diagnostic status. This apprehension was sometimes vindicated by dismissive professional responses.

*It was very nerve-wracking. I was very nervous that I would get the default answer of “get off the Internet”. […] then I brought up complex PTSD. And then they laughed and they're like, that's just too new and that's, no, that's not a thing, that's just fancy PTSD. And I'm like, no, I'm pretty sure that's an official diagnosis. [P3]*

##### Experiences of the Shift

In almost all cases framed as misdiagnosis or missed diagnosis, participants saw the diagnosis they held at interview as appropriate. Generally, participants were more comfortable with their current than previous diagnoses.

*life's just been a lot easier since I had a decent diagnosis. [P16]*

Relief was a common response to a diagnostic shift viewed as the correction of a previous misdiagnosis. Relief typically arose from feeling one had a better explanation of one's life and challenges.

*now that I have a different diagnosis, it actually makes me feel better. It's like, oh, you know, it's not my chemistry, there's nothing wrong with my chemistry, my brain, I'm not bipolar. There's nothing wrong with my personality, I'm not borderline. […] It's just like everything fit together for the first time, like it all made sense. [P13]*

For those who felt their correct diagnosis had been delayed, relief was mixed with regret and anger regarding “lost time” for understanding and intervention.

*I'm really pissed off. I've had to suffer all this time because no one was prepared to actually sit down and give me that diagnosis […] You're fighting a battle against depression, when you're fighting the wrong battle. […] You know, over the years of not understanding, that not making sense is just… well, it's quite traumatic. [P16]*

Participants who proactively sought a diagnostic shift described a sense of agency and empowerment when it was confirmed.

*I sought this one out myself, so it was like it was me driving it […] so it felt good how it happened. [P2]*

However, when diagnostic corrections were unexpectedly introduced by a clinician, immediate responses sometimes involved embarrassment or shame.

*I think for that diagnostic shift, there was like maybe… a sense of worry? Like it was the first one I had kind of experienced and I felt, almost a little bit embarrassed, like “oh my God, they think I was lying”. [P1]*

Negative responses to diagnostic shifts could be alleviated by clinical communication that involved the participant in diagnostic decision-making.

*he took the time to really, really explain […] the reason for the diagnostic shift, so why I may have presented as someone with BPD or why he believed that I didn't have it. […] So for me that was a really, really positive experience because it was the first time that I really understood what was going on for me. [P1]*

##### Consequences of the Shift

Numerous participants characterized a delayed or corrected diagnosis as a transformative moment, altering the trajectory of their lives.

*it's given me… I'd say like a new lease of life, but you know, I'm a lot more comfortable with myself. […] It was like somebody switched the light on. The end of the dark period […] that diagnosis is everything. [P16]*

Corrected diagnoses could transform lives through access to more effective treatment.

*one thing I just, that I think is so exciting about going through a new diagnosis is you've been treated for one thing, and now you're going to be treated for something else, and you have so many new treatment options and tools available to you. [P19]*

However, experience of misdiagnoses or missed diagnoses could compromise trust in both individual clinicians and standard diagnostic procedures.

*I agree with that diagnosis, but I think my main problem is like, I doubt it all the same, because I'm like, you've got it wrong before. [P6]*

Corrected diagnoses often sparked a retrospective reframing of one's life, shedding new light on past experiences. This autobiographical reflection could reconstitute one's self-concept, improving self-esteem or mitigating self-judgement.

*I didn't perceive my problems with that procrastination like attention problems. I mean, at this time I believed in a very, very heavy moral evaluation, like when you're not making an effort, you're not doing the work, then it's laziness, right? I grew up thinking of myself as lazy. Took some time to unlearn that. […] there was a lot of validation, definitely, a lot of stuff coming together, chronological. A lot of saved self-esteem, like you know, I'm not inferior, I'm not lazy. [P4]*

However, the self-disruption caused by a corrected diagnosis could also leave people feeling disoriented.

*I got another framework to look at myself. That's perhaps the most confusing part, you know, how the frameworks change and what parts of you you consider normal, your personality, and what are, you know, more of expression of some difficulties that are connected to diagnosis. So what parts of me were… yeah. That was really hard for me to reconcile. [P17]*

#### Evolving Diagnoses

##### Profile of the Shift

Certain diagnostic shifts were characterized as gradual evolution rather than abrupt conversion. Participants attributed these shifts to the accumulation of clinical evidence, which informed progressively more sensitive diagnostic decisions. The final diagnosis was simply the logical end-point of this process.

*I suppose I just saw the change as an evolution of the technical picture, do you know, so it's not like I thought the adjustment disorder was wrong because it wasn't at the time, it was fine, and you know it was part of the picture…. and then okay, so it was becoming a bit more clear that it was more than an adjustment disorder and then, you know, depression seemed to fit at that stage. [P23]*

##### Antecedents of the Shift

Diagnostic evolutions were typically precipitated by the emergence or disclosure of symptoms qualitatively different from the original clinical presentation. Sometimes these new symptoms were identified during a crisis episode, often involving hospitalization and/or new treating clinicians.

*I had got to the health center and they had said, well, you need to be in the hospital. When I was there, they also discovered some like psychosis and like paranoia and hallucinations. And so they changed my diagnosis to… they said I was having a mixed episode and they changed my diagnosis to bipolar II. [P10]*

Diagnoses could also evolve due to wider institutional developments. For example, multiple participants reported their diagnostic classifications had changed due to revision of diagnostic manuals, with the change of nomenclature experienced as perplexing.

*actually I hate that term […] emotionally unstable person, I absolutely hate it. If, I have to admit, if that had been the name of the disorder back when I had been diagnosed, I think I would have had a much stronger feelings of like being diagnosed with it. […] I would find it really invalidating to have been given a diagnosis of emotionally unstable personality disorder. [P22]*

##### Experiences of the Shift

Those who characterized their diagnostic shift as a progressive evolution generally saw this as a normative experience, or the way psychiatric diagnosis typically occurs. Personally, participants were largely indifferent to the shift.

*they usually start with adjustment disorder, it's kind of like what they start with before they have to put a diagnosis on it usually. So I don't know how long she had that but I just know that, you know, that's what they put when they first meet you. And then had, had me as depression and then generalized anxiety disorder and eating disorder not otherwise specified. [P18]*

Evolving diagnosis narratives often involved ambiguity around a participant's exact diagnostic status at particular points in their trajectory.

*he also said he thought I had borderline personality ‘tendencies', is the word he used with me, but so borderline personality tendency, I guess, I was diagnosed with and I never saw, like I never asked for written confirmation about the diagnosis, I just took it as it was. [P2]*

Such ambiguity was sometimes attributed to a perceived reticence among clinicians about giving diagnoses, which was not necessarily shared by service-users.

*I think that like my therapist […] he's uncomfortable with that diagnosis […] a lot of the frustration for me is that I think therapists have a lot of anxiety over things and over how patients might feel about diagnoses, but they never actually asked us how we feel about diagnosis, they assume. […] I didn't really care what my diagnosis is. [P22]*

##### Consequences of the Shift

The gradual, contiguous nature of diagnostic evolutions generally made these shifts comprehensible to participants, which fostered self-understanding.

*I could tell that there was something more going on, besides just the depression, and so I was like… Okay, that doesn't seem to fit so well anymore, but now, this new one, like okay, yeah. Like I read about it in the DSM and everything, yeah, this explains better what I was going through. [P10]*

Relative to corrected misdiagnoses, diagnostic evolutions were typically seen as less transformative, and did not necessarily alter treatment pathways.

*I think it just better explained what was going on. It was more… I mean it barely changed my– I don't think it changed my treatment at all, like, I was on the same medication, it was just a clarification. [P9]*

Diagnostic evolutions were usually not attributed to clinical error. Rather, the original diagnoses were seen as sensible given available information, and accurately updating in response to new evidence.

*these kinds of issues that I have in emotional regulation could be easily described by both ADHD and PTSD […] I think that it made sense to diagnose me with borderline personality disorder before they got to know my whole history. [P17]*

Evolving diagnoses therefore did not provoke the same crises of clinical trust evident in mis- or missed diagnoses. Trust was particularly promoted when diagnostic evolutions were negotiated collaboratively and transparently.

*I switched therapists and it was kind of like, well it's been a while, let's talk about this, like. Well she saw me for a while and she's like, I'd like to put down generalized anxiety, that feels like it's really just kind of the fit for you like at this point. Like how do you feel about that? [P21]*

#### Proliferating Diagnoses

##### Profile of the Shift

A narrative of proliferating diagnoses applied to a subset of participants, who received between 5 and 10 diagnoses across successive diagnostic episodes. These participants sometimes experienced retractions of certain diagnosis. However, several also reported an understanding that multiple successive diagnoses remained on their clinical records, despite some ceasing to require active clinical attention.

*no one has taken off the unspecified or OCD despite my clear signs of autism, ADHD, depression and anxiety. I still have the two unspecified and OCD as open diagnoses. They're not locked down, they're not removed either. Just nobody has touched since I got them. […] They mostly have just ignored it. And that is a bit weird I think. [P12]*

##### Antecedents of the Shift

Typically, those who experienced proliferating diagnoses had cycled through multiple different clinicians. A new clinician could disagree with former diagnoses, and either replace or supplement them with new diagnoses.

*I switched back to another psychiatrist that was recommended to me by my therapist. After meeting with him, he did not agree with borderline, the BPD diagnosis, so he actually went back to bipolar II. [P14]*

Participants reported that clinicians often failed to explain how a new diagnosis related to prior diagnoses, which were left untouched on their clinical records. However, it was also possible for prior diagnoses to be explicitly retracted by a new clinician, or for diagnoses to be “lost” in the move between clinicians.

*the borderline just kind of fell off when I changed doctors […] I think the only reason that actually left my file is that I changed doctors and I don't think they got the notes from my previous doctor. [P9]*

While self-sought diagnostic shifts were less common in narratives of proliferating diagnoses, some participants reported that certain clinicians afforded them considerable agency in determining their diagnosis.

*she just said do your own research kinda, see how you feel about it, see if it fits and then pretty much like if you feel like it fits, then we'll go with that, and if you don't feel, like if it's no, we won't. So it was very strange to me that, like I was almost put in charge of diagnosing myself. [P14]*

This was not universal; during other diagnostic episodes, participants felt like passive observers of professionals' decisions.

*she wrote like borderline, question mark and then that's what they ran with. […] it was very confusing because there was no, like I couldn't see any of the documents and I couldn't like talk to her about it and she didn't talk to me about it. So yeah, just kind of felt like it came out of nowhere. [P5]*

##### Experiences of the Shift

Like evolving diagnoses, proliferating diagnoses were associated with some ambiguity. Participants sometimes struggled to remember or expressed uncertainty about points in their diagnostic trajectory.

*I was diagnosed with major depression, manic depression, I don't know. At one stage they diagnosed me as three different types of depression, personality disorders, complex PTSD, lots of different labels that they just kept throwing at me and every time I'd say there was something else wrong with me they'd just label it with… personality disorder was a big one for a while. [P16]*

Some participants positioned one particular diagnosis as a landmark revelation, describing relief when that was confirmed.

*For me, the diagnosis was simply a confirmation that allowed me to say to other professionals that I am autistic without being told “you can't possibly know that” […] What the diagnosis did was change how I felt about myself—for the first time, I was able to truly forgive myself for executive functioning difficulties, social difficulties, and extreme clumsiness. [P26]*

However, a long list of regularly changing diagnoses caused confusion and frustration.

*I'd go away and think, okay well yeah, I'm depressed and I'm like this because this happened and trying to sort of identify, trying to put the jigsaw together but being given the wrong pieces, never getting anywhere. And when you'd go to them again, they'd give you a different piece. And that wrecks your head for six months, it just doesn't fit together. And then you'd go back and they'd give you a different puzzle, and you go away to try and get together all the new puzzle, but it doesn't make sense, it doesn't make anything better. [P16]*

##### Consequences of the Shift

Having one's diagnosis constantly mutate could be demoralizing. Several participants internalized their diagnostic complexity to infer that they were uniquely damaged.

*experiencing a series of diagnostic shifts and like none of them fully feeling like they made sense really made me feel like ‘oh I'm just so broken, like they just can't figure out like what's going on', you know? Like maybe there's a hopeless cause. [P1]*

Socially, frequent changes to one's diagnosis denied people a clear diagnostic group with which to identify, and were difficult to explain to others.

*I did tell some of the closest people in my life […] because it was relevant at the time but I'm not sure I would continue to tell about it. I think… I'm not sure, perhaps it was my impression that it made them a little bit more skeptical toward me, some of them. Because it was, you know, a huge change from borderline to, you know, PTSD and ADHD, yeah, ADHD… perhaps like if I was shoplifting the diagnosis, you know. They didn't say that but I kind of…. They were confused about the changes. They didn't really understand why. [P17]*

Experience of proliferating diagnoses often undermined clinical trust. Exposure to differences of opinion across professionals and time cultivated doubt in the validity of psychiatric diagnoses, making them seem random or arbitrary. Some withdrew from clinical settings as a result.

*they just kept throwing diagnoses at me that… I was kind of along for the ride, like I started to lose trust in them, because the longer I went in the more, the more like big diagnoses they were giving me. […] at that point, I had just officially given up completely, and like, I will figure out my mental health on my own. I'm not going back to a doctor. [P3]*

### Diagnosis-Specific Factors

Similarities and disparities in participants' experiences were not entirely attributable to whether diagnostic shifts were characterized as proliferating, evolving, or mis-/missed diagnoses. Cutting across all three interpretations, participants raised the significance of the exact diagnostic labels involved. The second thematic dimension captures how responses to a diagnostic shift hinged on the unique meanings ascribed to the specific diagnoses gained and lost. Table 2 summarizes how in participants' accounts, diagnoses were differentiated by four key factors: severity, stigma, personal associations, and communities.

**Table 2:** *Diagnosis-specific factors*: pathways through which they influenced responses to diagnostic shifts.

	**More positive responses to shift if**	**More negative responses to shift if**
Severity	Perceived reduction in diagnostic severity	Perceived increase in diagnostic severity
Stigma	Decreased stigma exposure	Increased stigma exposure
Personal associations	Positive associations with new diagnosis in individual's personal networks	Negative associations with new diagnosis in individual's personal networks
Communities	Access to valued new identities	Disruption of valued old identities

#### Severity

Certain diagnoses were seen as more clinically serious than others. For example, psychotic and personality disorders were viewed as more pervasive, permanent, and disruptive than affective or anxiety disorders. Revised diagnoses perceived as less bleak than the originals elicited relief.

*really relieved because I felt like, oh, this means that I'm not going to be depress–like depressed my whole life. Like I can, I do have, my body does have the ability to feel happy and joy and feel resilient. And even if it's not in that big way of the hypomanic feeling, like I was really excited that I wasn't just going to be depressed forever. And I knew that depression was going to be part of, it's going to be part of my life forever, but I really felt just this huge sense of relief that that wasn't going to be the only feeling. [P19]*

Conversely, diagnoses seen to represent increases in clinical severity prompted despair.

*something like depression and anxiety you hear about all the time. And then obviously like the portrayals of people with psychosis in the media are like always a lot more dramatic and I kind of had, in my head, that that was like something totally incurable and that I was just crazy and that like nobody could relate to me. And that basically my prognosis was really bad because of that diagnosis. So I think it was– is more disheartening. [P1]*

Yet despite such discouragement, some participants found validation in clinical confirmation that their challenges were more serious (and hence achievements more significant) than originally acknowledged.

*I mean the prognosis for it, schizoaffective is definitely worse so it's kind of like… when I first heard that diagnosis applied to me, I was sort of like, “Oh, like that's a disappointment.” […] it's worse, but then it's also like, okay I'm–I'm overcoming this so I feel like that's sort of like a sense of… success I guess? [P10]*

#### Stigma

The issue of diagnosis-specific stigma arose in all interviews. Narratives implied a “stigma hierarchy” with some diagnoses perceived as more stigmatizing than others. Participants welcomed shifts that reduced stigma exposure and struggled with shifts to diagnoses carrying greater amounts of stigma.

*there's so much stigma attached to BPD and I really wasn't, wasn't comfortable having that diagnosis. And say even now, like, I like to make the point that I was undiagnosed with that later. Like I think there's just so much stigma attached to that particular disorder. [P1]*

Participants were acutely aware of the stereotypes attached to new diagnoses, and abhorred the projection of these stereotypes onto oneself.

*it was awful. It was like everything, everything I had worked toward in recovery to understand that there isn't this black bad person inside me […] then to sit with people who are misinterpreting you every hour of every day and thinking you're being bold or however they think of borderline people. And you know, even when I'd be trying to eat my meals, I'd feel like, oh God they're looking at me, like, ‘ugh would she ever just eat, she hasn't got a real problem with food', you know, that she's just attention-seeking or whatever, like you know. And yeah, so it was really, really, really disturbing and it kind of took away my motivation to get well. [P25]*

There were some suggestions that stigma also affected clinical practice around particular diagnoses. For instance, four participants reported a discovery that a personality disorder diagnosis had been ascribed to them without their knowledge, suspecting clinicians wished to spare them the presumed stigma.

*I did an outpatient programme which was when they told me I was diagnosed with borderline because I didn't know before. When the psychologist diagnosed me, I just, she didn't tell me anything or whatever, so it was in my medical record […] I was like […] so shocked. I was like, wait what? And no one thought to talk to me about that? […] I kind of had the feeling that, like, I was the last to know. And like yeah, made me feel like a little bit infantilised. [P5]*

#### Personal Associations

Beyond sociocultural stigma, certain diagnoses could also hold personalized meanings that mediated responses to diagnostic shifts. For example, shifts to diagnoses that were shared with relatives confronted participants with a vivid exemplar of the daily reality of living with that diagnosis.

*my mom and I were shopping for shoes and she was just like, yeah your dad has that. And I'm like, what? She told me these horrible stories about what it was like to be married to him—they're divorced—what it was like to be married and his like psychotic breaks with reality. I was like, what? I still remember that, I just started crying, I'm awful. [P13]*

Diagnoses could also hold idiosyncratic implications in occupational contexts. For example, one health professional was particularly uneasy about her diagnosis of ADHD, which among several diagnoses uniquely jeopardized her professional reputation.

*I would probably have the most stigma internally around ADHD. That's the one I don't disclose. [P23]*

Experiences of a diagnostic shift were additionally shaped by the associations friends and family held with a particular diagnosis. Participants reported both positive and negative social responses to disclosure of a diagnostic shift, depending on the meaning a diagnostic label held within one's social circle.

*I talked to my spouse at the time. […] he was very much like, well only the military gets PTSD. So at that point, I just learned to just keep it all to myself. [P3]*

*the people I hang out with, they don't give a… they don't give a sh* ^*^ *t about BPD. [P8]*

#### Communities

By re-classifying individuals from one category to another, revised diagnoses could both open and close access to communities of similarly diagnosed others. Such diagnostically-bounded communities were often platforms for valued social identities. This was particularly salient for neurodevelopmental diagnoses such as ASD and ADHD.

*Depression is weird in a way, that if I had some other friends who had depression, I didn't relate to them at all. […] it's like everybody has a different depression. And with ADHD it's the exact opposite. […] It's crazy how easily we connect with ADHD. [P4]*

Diagnostic shifts that presented opportunities to form bonds with similar others were experienced as emotionally rewarding and practically helpful.

*the point when I finally saw other people that were dealing with similar things—that really informed me a lot. So I think that that's very helpful. [P15]*

Conversely, participants struggled with diagnostic shifts that erased the shared classification at root of valued social bonds.

*I like contacted the service-user network and like went along there and also […] my mom was like, privately she told me that […] that was the same diagnosis that she had had. And so I was like, oh everything's making sense and I like started to feel like, oh this is like something that fits for me. […] I got a call from her [doctor] and she was like, “oh my supervisor has reviewed my diagnosis and she doesn't think it's like, it's right for you” […] it was really, really difficult […] it was just like invalidation. [P24]*

However, embrace of the social identities attached to particular diagnoses was not universal. Diagnostic shifts that challenged existing identities, or that led people into social settings where they felt they did not belong, were difficult to absorb.

*it can take away… your identity becomes ingrained with the diagnosis and then, when you are reconsidering another one it's like, well then, who am I? You lose that sense of self. [P13]*

## Discussion

Though the socio-emotional significance of diagnostic classifications and the frequency of shifts between such classifications are widely acknowledged, minimal research has explored how diagnostic shifts are subjectively experienced by service-users. The current study represents the first to investigate how adult service-users make sense of diagnostic shifts and their impacts on one's life. Interviews with people with first-hand experience of diagnostic shifts revealed common features in their narratives, as well as salient points of divergence. Many of these patterns mapped onto a typology of interpretations of diagnostic shifts: diagnostic shifts were experienced differently, depending on their framing as evolving, proliferating or mis-/missed diagnoses. Also critical in determining diagnostic shift experiences were the specific diagnoses involved, and the unique meanings these classifications held in participants' personal and social ecosystems.

Results contribute to the literature by revealing the importance of the interpretations service-users develop to understand the reasons behind their diagnostic shifts. The diagnostic shifts that saw smoothest adjustment were those characterized as evolving diagnoses. As these shifts were seen to follow from the emergence of new symptoms or accumulation of clinical evidence, they did not require nullifying previous diagnoses as “wrong.” This escape from binary “correct/incorrect” framings softened these shifts' impact: evolving diagnoses were not typically framed as overhauling one's life, though could yield subjective benefits in increasing self-understanding. In contrast, diagnostic shifts attributed to clinical errors in missing or misdiagnosing symptoms were cast as transformative in participants' lives. Mis- or missed diagnoses' perceived correction was typically welcomed by participants, both for emotional validation and alignment of more effective treatment. However, consistent with previous research (8, 32, 33), delayed or corrected diagnoses could leave people feeling disoriented and regretful. They could also shake confidence in individual clinicians and mental health systems more generally. With prior evidence showing trust is an important factor in treatment engagement (34), this may undermine long-term recovery prospects.

Narratives of proliferating diagnoses had perhaps the most negative implications for self-concept, social relations and clinical trust. Participants who had cycled through many different diagnoses, typically delivered by numerous clinicians, were confused and demoralized by their experiences. Whereas some participants adapted by fixating on one particular diagnosis as a watershed revelation, others developed an impression of diagnoses as arbitrary labels. While this could be interpreted as an appropriately critical perspective on the ontological status of categorical diagnoses (20, 22), it often cultivated therapeutic pessimism (35), with participants inferring that the complexity of their difficulties confounded conventional clinical frameworks.

Results also showed how, beyond the distinctive patterns associated with the various interpretations of diagnostic shifts, experiences were contingent on the specific diagnoses involved. Shifts from one diagnosis to another brought relative advantages and disadvantages, depending on the unique meanings attached to each. Shifts connoting an increase in severity caused trepidation, while reductions in perceived severity caused relief. Participants welcomed re-classifications that reduced their stigma exposure, and resisted diagnoses that triggered pejorative stereotypes. Some compensation for stigma was available in the marshaling of diagnoses as bases for communities and social identities, but participants regretted shifts that removed them from valued communities. Moreover, specific diagnoses could have highly personalized associations within participants' social networks, which also mediated responses to diagnostic shifts. While much previous research has demonstrated the diagnostic specificity of sociocultural representations of mental illness (17–19), this is the first study to demonstrate how this variability in meaning shapes the experiences of those whose diagnostic classifications change over time.

These findings regarding the diagnostic specificity of adults' responses to clinical reclassifications echo those from the only previous study of lived experiences of diagnostic shifts, which focused on children and their parents (8). As in the present study, families' responses to diagnostic shifts hinged on the specific diagnoses involved, and the unique implications of those labels for a young person's self-concept, social relations, and experience of services (8). The current adult-focused study advances beyond this previous research to also focus attention on the key role played by the particular interpretations that service-users apply to their own cases of diagnostic shift. In adults, diagnostic shifts trigger active cognitive work to understand these clinical decisions, and the conclusions drawn shape diagnostic shifts' implications for therapeutic engagement, clinical trust and self-understanding.

The research advances sociological understandings of psychiatric diagnosis by highlighting the variability of diagnostic experiences. Importantly, variability occurs both between and within participants: the same person can experience successive diagnostic episodes very differently, depending on their subjective and social contexts. Results challenge a strongly deterministic view of diagnostic labeling, which construes diagnostic labels as immovable marks that restrict people's lives *via* a “self-fulfilling prophecy” effect (36). Not only do diagnoses fluctuate in clinical practice, but individuals relate to them in diverse ways, with some welcoming the solidity of binary classifications, others viewing diagnoses as fluid “best-guess” approximations of their experiences, and others rejecting their validity entirely. Future research should consider that construing service-users as active participants in, rather than passive recipients of, psychiatric diagnosis may be crucial for understanding the real-world significance of diagnostic labels.

Findings raise implications for clinical practice. First, clinical communication about diagnostic shifts should be transparent and inclusive. Throughout the data, participants who were afforded meaningful input in diagnostic decision-making found it easier to adjust to their re-classification. Diagnostic shifts that were sudden and unanticipated often elicited turbulent receptions, even if participants adapted to that re-classification in time. Second, clinicians should take time to explain and contextualize the reasons for a diagnostic shift. The same diagnostic trajectories were framed differently across participants, with some considering their original diagnosis a factual error and others attributing it to incomplete clinical information. Since shifts framed as evolving diagnoses were typically easier to assimilate, and posed fewer threats to therapeutic engagement, clinicians may benefit from explaining the rationale for the original diagnosis, and the changing circumstances that led to its revision. Third, participants' reports suggested that accumulation of multiple disconnected diagnoses is often linked with a lack of continuity between treating clinicians. Clinicians assessing a service-user with prior clinical engagements should determine their previous diagnoses, and explicitly confirm or rebut each. Previous documentary research with medical records confirms that historic diagnoses can be left untouched on medical records despite ceasing to inform clinical pathways (37); this study shows how this can cause confusion and frustration for service-users. Fourth, consideration of diagnoses' differential implications in terms of severity, stigma and social opportunities can help clinicians anticipate service-user responses to particular diagnostic shifts. Prior research shows that a trusting therapeutic alliance can buffer the impact of diagnoses that may be perceived as “bad news” (38). Supporting service-users to process the global implications of the shift for their life, beyond purely clinical repercussions, may ease the transition.

### Strengths and Limitations

The project collected in-depth data from an international cross-section of participants, representing a respectable sample-size for a qualitative study (39). However, reliance on self-selecting English-speaking participants meant all were from Europe or North America. There are cross-cultural differences in the meanings attached to psychiatric diagnoses (18, 40), and this study does not illuminate how diagnostic shifts are experienced in non-western or low/middle-income countries. At the same time pooling participants from seven countries within one dataset meant cross-country differences went unexamined. Systematic international comparisons represent fertile areas for future research; for instance, perhaps the greater privatization of US mental healthcare makes self-sought diagnostic shifts more common than in the UK's National Health Service. Future research should also investigate how diagnostic shift experiences are inflected by socio-economic status and race/ethnicity: ethnic minorities tend to receive more severe diagnoses (41), and may be less likely to experience diagnostic shifts (2). The current sample was biased toward women, who typically experience different mental health difficulties than men (42) and may be more likely to undergo diagnostic shifts (43). Expansion of data on men's experience of diagnostic shifts should be a research priority. Furthermore, the opt-in recruitment method, open to all diagnostic trajectories, may have led to over-representation of some diagnoses (e.g., BPD) relative to their population prevalence. However, the aim of qualitative research is not statistical representativeness (23), and inclusion of diverse diagnostic classifications had benefits in sharply revealing the importance of diagnosis-specific meanings. The latter finding highlights the need for future research pursuing closer analysis of specific diagnostic transitions. For instance, several participants reported a shift from BPD to PTSD; an exclusive focus on this cohort may reveal distinctive features of this experience, with applications for a more defined area of clinical practice.

The data collection approach generated rich data. By asking people to relate their story in their own words, narrative interviews afford participants control over discussion, serving both ethical and empirical validity. However, a more structured interview schedule may promote consistency in material covered and facilitate incorporation of frequency data within the qualitative analysis. The current qualitative analysis aimed to map the range of experiences present in the sample, not to generalize findings beyond this dataset. Future quantitative research could systematically investigate whether different service-user interpretations of diagnostic shifts reliably correlate with particular antecedents and consequences. While this critical realist analysis did not consider the factual accuracy of participants' accounts, deeming this immaterial to understanding their subjective implications, future research could also compare participant narratives with clinician perspectives on the causes, direction and timing of diagnostic shift events. For instance, it would be interesting to know whether clinicians share service-users' judgements on whether particular diagnostic shifts were due to clinical error, or the clinical communication strategies that support interpretations of diagnostic shifts as evolving diagnoses.

## Conclusion

The complexities of diagnostic shifts are largely a byproduct of standard categorical systems of diagnosis; shifts would be softened or eradicated by formulation or dimensional approaches to clinical assessment, which obviate the need for binary categories (22). However, dimensional systems remain some distance from widespread clinical application, and non-diagnostic approaches may be resisted by some clinicians and service-users, who appreciate the clarity of categorical diagnoses (12). Diagnostic shifts are therefore likely to remain part of the foreseeable future of clinical practice. The current research offers first insights into the factors that may influence lived experiences of diagnostic shifts, suggesting they are contingent on service-users' interpretations of the reasons behind the shift, and the unique meanings attached to the specific diagnoses in question. Further research that advances understanding of the prevalence and predictors of variable experiences of diagnostic shifts is critical for informing sensitive clinical practice and communication.

## Data Availability Statement

The datasets presented in this article are not readily available because full interview transcripts cannot be made public in order to preserve participants' anonymity. Raw extracts from transcripts can be obtained on request to the corresponding author. Requests to access the datasets should be directed to cliodhna.oconnor1@ucd.ie.

## Ethics Statement

The studies involving human participants were reviewed and approved by University College Dublin Human Research Ethics Committee (ref HS-21-03-O'Connor). The patients/participants provided their written informed consent to participate in this study.

## Author Contributions

CO'C contributed to conception and design of the study and wrote the first draft of the manuscript. CO'C and CS collected the data. CO'C, CS, and CY contributed to the qualitative analysis. All authors contributed to manuscript revision, read, and approved the submitted version.

## Funding

This research was supported by a grant from University College Dublin awarded to CO'C. Funders had no role in the design of, conduct of or decision to publish the research.

## Conflict of Interest

The authors declare that the research was conducted in the absence of any commercial or financial relationships that could be construed as a potential conflict of interest.

## Publisher's Note

All claims expressed in this article are solely those of the authors and do not necessarily represent those of their affiliated organizations, or those of the publisher, the editors and the reviewers. Any product that may be evaluated in this article, or claim that may be made by its manufacturer, is not guaranteed or endorsed by the publisher.

## Footnotes

^1^One participant requested that their interview take place over email due to difficulties with oral communication; this preference was accommodated.

^2^Verbatim quotes from interviews are identified according to anonymized participant numbers.
