# Supplementary material for: How Does It Feel to Have One's Psychiatric Diagnosis Altered? Exploring Lived Experiences of Diagnostic Shifts in Adult Mental Healthcare
Source: Front Psychiatry. 2022 Feb 11;13:820162. doi: 10.3389/fpsyt.2022.820162 (PMC8873081; doi:10.3389/fpsyt.2022.820162)
Supplement: Supplementary file 1 [file Table_1.pdf]

Supplementary Material

Table S1. Participants' self-reported diagnostic trajectories

Participant number	Diagnostic trajectory
P1	Depression, Generalised Anxiety Disorder (GAD) → + Psychosis → + Obsessive Compulsive Disorder (OCD) → + Borderline Personality Disorder (BPD) → + Post Traumatic Stress Disorder (PTSD), - BPD
P2	Gender Dysphoria, BPD → + Attention Deficit Hyperactivity Disorder (ADHD) → + Autism Spectrum Disorder (ASD)
P3	Anxiety Disorder (with panic attacks) → + ADHD → + PTSD, + Depression → + Bipolar Disorder I → + GAD → + Schizoaffective Disorder, - Bipolar Disorder I → - Anxiety Disorder, - ADHD, - GAD, - Schizoaffective Disorder, + Gender Dysphoria, + Other Specified Feeding or Eating Disorder (OSFED), + OCD, + Major Depressive Disorder (MDD)
P4	Depression → + ADHD → - Depression
P5	Tourette Syndrome → + MDD, + GAD → + BPD → + ASD
P6	Depression → + BPD/Emotionally Unstable Personality Disorder (EUPD) → + Complex PTSD, - BPD/EUPD
P7	ADHD → + Depression → + Bipolar Disorder, - Depression → + PTSD
P8	Substance Use Disorder → + BPD → - BPD
P9	Depression → + Bipolar Disorder II, + BPD, - Depression → + Bipolar Disorder I → + Schizoaffective Disorder
P10	MDD, GAD, Social Anxiety Disorder → + Bipolar Disorder II, - MDD → + Bipolar Disorder I, - Bipolar Disorder II → + Schizoaffective Disorder, - Bipolar Disorder I
P11	Psychotic Disorder Not Otherwise Specified → + PTSD → + Schizoaffective Disorder (Bipolar Type)
P12	Tourette Syndrome, Unspecified Developmental Disorder → + OCD → + ASD → + ADHD → + Depression, + Anxiety Disorder
P13	Bipolar Disorder → + GAD (with panic attacks) → + BPD → + Complex PTSD, - Bipolar Disorder, - BPD
P14	MDD, GAD → + Bipolar Disorder II, + GAD → + ADHD → + BPD, - Bipolar Disorder II → + Bipolar Disorder II, - BPD
P15	Depression → + Social Anxiety Disorder → + Personality Disorder Not Otherwise Specified → + Complex PTSD
P16	Double Depression (Dysthimia, Recurrent Depressive Disorder), Substance Use Disorder, Personality Disorder Not Otherwise Specified (Mixed Personality Disorder with Borderline and Dissocial and Paranoid Traits) → + Bipolar Disorder II/Cyclothymia, + EUPD, - Substance Use Disorder → + Complex PTSD
P17	Trichotillomania → + Neurosis → + Complex PTSD → + Borderline Personality Disorder, + Stress Reaction → + ADHD, - BPD
P18	Adjustment Disorder → + Depression, + GAD, + Eating Disorder Not Otherwise Specified (EDNOS) → - GAD → + OSFED, - EDNOS → + Avoidant Restrictive Food Intake Disorder (ARFID), - OSFED → - ARFID
P19	MDD, GAD → + Bipolar Disorder II, - MDD
P20	MDD → + GAD → + ADHD
P21	ADHD, manic depression → + Bipolar Disorder → + BPD → + GAD
P22	BPD, Complex PTSD, EDNOS, MDD, GAD → + ADHD → - EDNOS, - BPD
P23	Adjustment Disorder → + MDD, + GAD, + PTSD → + ASD → + ADHD
P24	MDD → + GAD → + EUPD → - EUPD
P25	Anorexia Nervosa/Bulimia Nervosa → - Bulimia Nervosa → + BPD → - BPD
P26	Depression → + GAD, + PTSD → + Bipolar Disorder → - Bipolar Disorder → + ASD, + ADHD, + Dyspraxia, + Dysthymia, - Depression
P27	ADHD → + ASD, + MDD, + Anxiety Disorder Unspecified

Note: Arrows (→) separate diagnostic episodes, plus signs (+) indicate the addition of a diagnosis, minus signs (-) indicate the retraction of a diagnosis. In accordance with the critical realist research approach, Table S1 presents the diagnostic terminology that was reported by participants and/or their supporting documentation,

which did not always align with accepted diagnostic nomenclature (e.g. several participants reported receiving a diagnosis of 'Depression' rather than the more formal 'Major Depressive Disorder').
